# Supplementary material for: The Chengjiang Biota inhabited a deltaic environment
Source: Nat Commun. 2022 Mar 23;13:1569. doi: 10.1038/s41467-022-29246-z (PMC8943010; doi:10.1038/s41467-022-29246-z)
Supplement: Supplementary file 1 — Supplementary Information [file 41467_2022_29246_MOESM1_ESM.pdf]

The Chengjiang Biota inhabited a deltaic environment
Supplementary Information

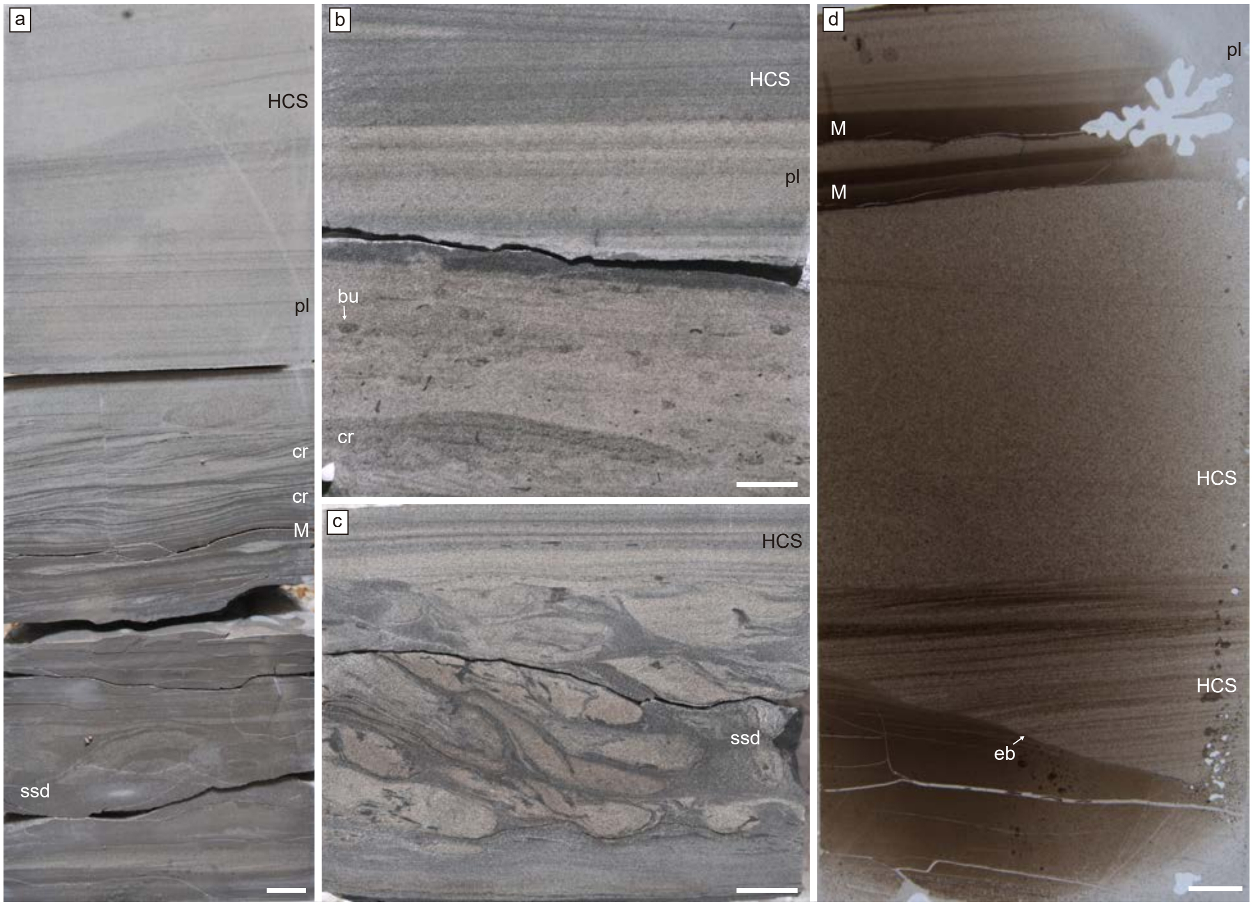

Supplementary Figure 1: Core photographs (a-c) and a thin section image (d) of oscillatory flow deposits of the Yu'anshan Formation. Scale bars are 1 cm. a) Interbedded mudstone (M) associated with hummocky cross-stratified (HCS), parallel-laminated (pl) and current-ripple cross-laminated (cr) sandstone. Soft-sediment deformation structures (ssd) are observed in the heterolithic bedsets. b) Bioturbated mottled intervals with undetermined burrows (bu) recording infaunal colonization of a current-ripple cross-laminated sandstone (cr). On top, hummocky cross-stratified (HCS) and parallel-laminated (pl) sandstone occur. c) Soft-sediment deformation structures (i.e. ball and pillow, ssd) embedded in the underlying mudstone. Hummocky cross-stratified sandstone (HCS) occurs on top. d) Gutter casts with erosive base (eb), infilled by hummocky cross-stratified (HCS) sandstone, and capped by mudstone intervals (M).

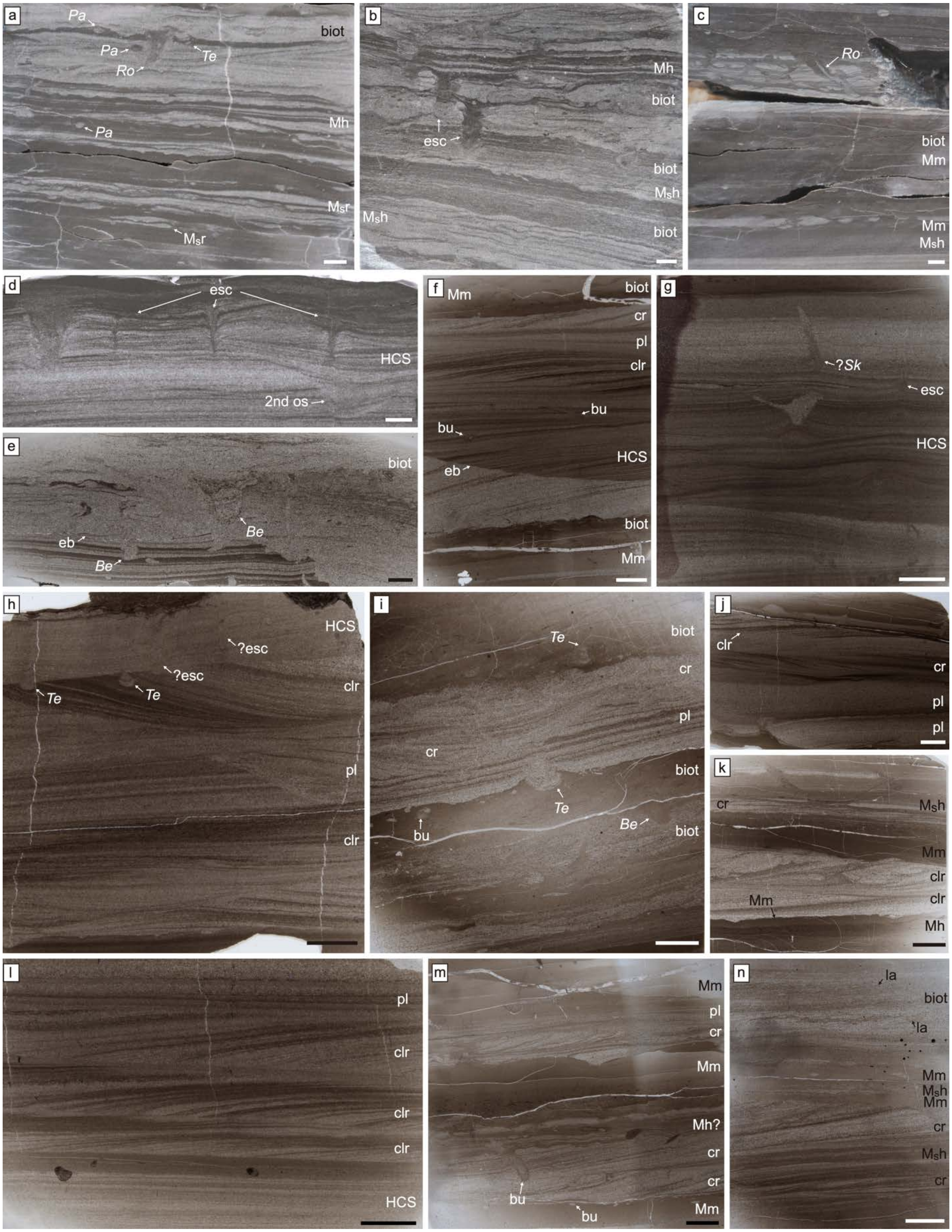

Supplementary Figure 2: Core (a-e) and thin sections (f-n) photographs of hyperpycnal flow deposits of the Yu'anshan Formation. Scale bars are 0.5 cm. a) Current-ripple cross-laminated (M_sr) and parallel-laminated (Mh) very fine-grained sandstone and mudstone showing discrete

trace fossils such as *Palaeophycus* isp. (*Pa*), *Planolites* isp. (*Pl*), *Rosselia* isp. (*Ro*), and *Teichichnus rectus* (*Te*), locally overprinting undifferentiated bioturbation mottling (biot). b) Parallel-laminated, coarse mudstone (M_sh) and fine and coarse mudstone (Mh) with escape trace fossils (esc) and bioturbated intervals (biot). c) Massive (Mm) and parallel-laminated (M_sh) fine to coarse mudstone, and current-ripple cross-laminated, very fine-grained sandstone with *Rosellia* isp. (*Ro*), whereas bioturbated intervals (biot) occur in the mudstone. d) Escape trace fossils (esc) in hummocky cross-stratified, very fine-grained sandstone (HCS) delineated by second-order surfaces (2nd os). e) *Bergaueria* isp. (*Be*) in heterolithic beds and within an erosive-based (eb), gutter cast filled by very fine-grained sandstone with undetermined bioturbation (biot). f) Massive mudstone (Mm) with bioturbated tops (biot), intercalated with very fine-grained sandstone with hummocky cross-stratification (HCS), current ripples (cr), climbing ripples (clr), and parallel lamination (pl). Erosive bases (eb) delineate ripple beds. Isolated undetermined burrows (bu) occur within the sandstone. g) Several amalgamated, hummocky cross-stratified, very fine-grained sandstone beds (HCS) with ?*Skolithos* isp. (?*Sk*) and poorly defined escape trace fossils (?esc). h) Climbing ripple cross-laminated (clr), parallel-laminated (pl), and hummocky cross-stratified (HCS) very fine-grained sandstone with *Teichichnus rectus* (*Te*) and poorly defined escape trace fossils (?esc). i) Interbedded bioturbated mudstone (biot) and very fine-grained sandstone with parallel- (pl) and current ripple cross-laminated very fine-grained sandstone (cr). Discrete trace fossils such as *Teichichnus rectus* (*Te*) and *Bergaueria* isp. (*Be*). j) Parallel- (pl), current ripple (cr), and climbing ripple (clr) cross-laminated very fine-grained sandstone. k) Massive mudstone (Mm) at the base, followed by current ripple (cr) cross-laminated very fine-grained sandstone, massive mudstone, and parallel-laminated coarse and fine mudstone (M_sh). Locally, burrows (bu) are observed. l) Composite beds of hummocky cross-stratified (HCS), climbing ripple cross-laminated (clr) and parallel-laminated (pl) very fine-grained sandstone. m) Interbedded current ripple cross-laminated (cr), and parallel-laminated (pl) very fine-grained sandstone with burrows, and parallel-laminated (Mh) and massive (Mm) mudstone. n) Interbedded very fine-grained sandstone with current ripples (cr) and low-angle cross-lamination (la), parallel-laminated coarse and fine mudstone (M_sh), and massive mudstone (Mm). The sandstone is locally bioturbated (biot).

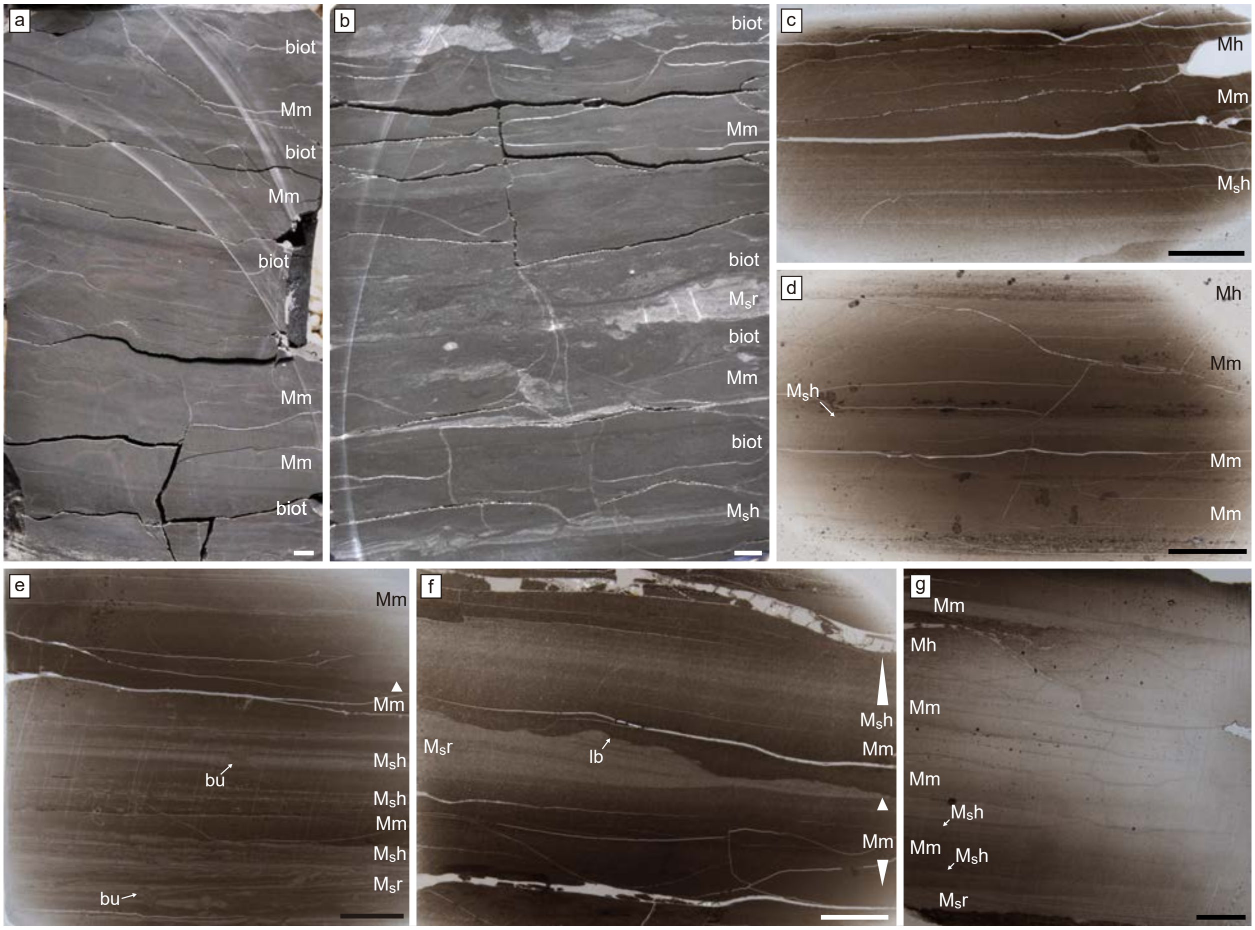

Supplementary Figure 3: Core (a, b) and thin sections (c-g) photographs of the wave-enhanced fluid mud deposits. Scale bars are 0.5 cm. a, b) Interbedded massive mudstone (Mm), and current-ripple cross-laminated (M_sr) and parallel-laminated (M_sh) coarse and fine mudstone with bioturbated intervals at the top (biot). c) Intercalated massive (Mm) and parallel-laminated mudstone (Mh), and parallel-laminated coarse and fine mudstone (M_sh). d) Massive (Mm) and parallel-laminated mudstone (Mh), locally showing coarse mudstone laminae (M_sh). e) Massive mudstone (Mm) intercalated with current ripple cross-laminated coarse mudstone (M_sr), and parallel-laminated coarse and fine mudstone (M_sh) with burrows (bu). f) Massive mudstone (Mm) with loaded base (lb), interbedded with normally and inversely graded (white triangles), and current-ripple cross-laminated (M_sr) coarse to fine mudstone. g) Composite bed showing current ripple cross-laminated coarse mudstone (M_sr), followed by parallel-laminated coarse and fine mudstone (M_sh), massive (Mm), and parallel-laminated (Mh) mudstone.

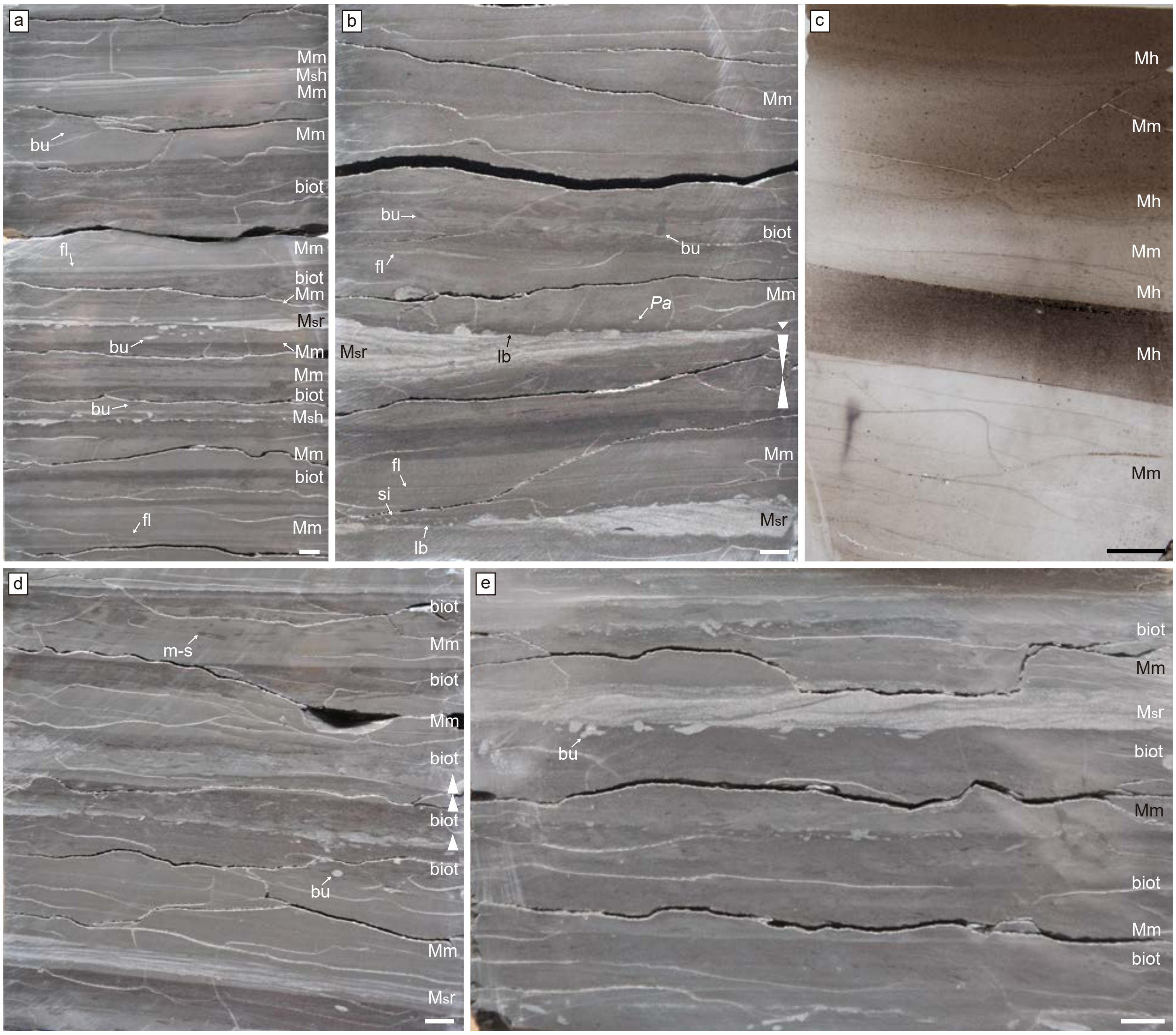

Supplementary Figure 4: Core photographs (a, b, d, e) and a thin section image (c) of the plug flow and low-density turbidity current deposits. Scale bars are 0.5 cm. a, b) Interbedded massive mudstone (Mm) with local faint lamination (fl), and current-ripple cross-laminated (M_sr) and parallel-laminated (M_sh) coarse to fine mudstone. Bioturbated intervals (biot) occur on top of the beds, whereas *Palaeophycus* isp. (*Pa*) discrete burrows (bu) can be delineated within the beds. Locally, inverse and normal gradation (white triangles), loaded bases (lb) and sandstone intraclasts (si) can be observed in the mudstone. c) Thin section of interbedded massive (Mm) and parallel-laminated mudstone (Mh). d) Massive (Mm) and normal-graded (white triangles) mudstone intercalated with current-ripple cross-laminated fine to coarse mudstone (M_sr). Bioturbation (biot) can be observed towards the top. Trace fossils include mantle-and-swirl structures (m-s) and undetermined discrete burrows (bu).

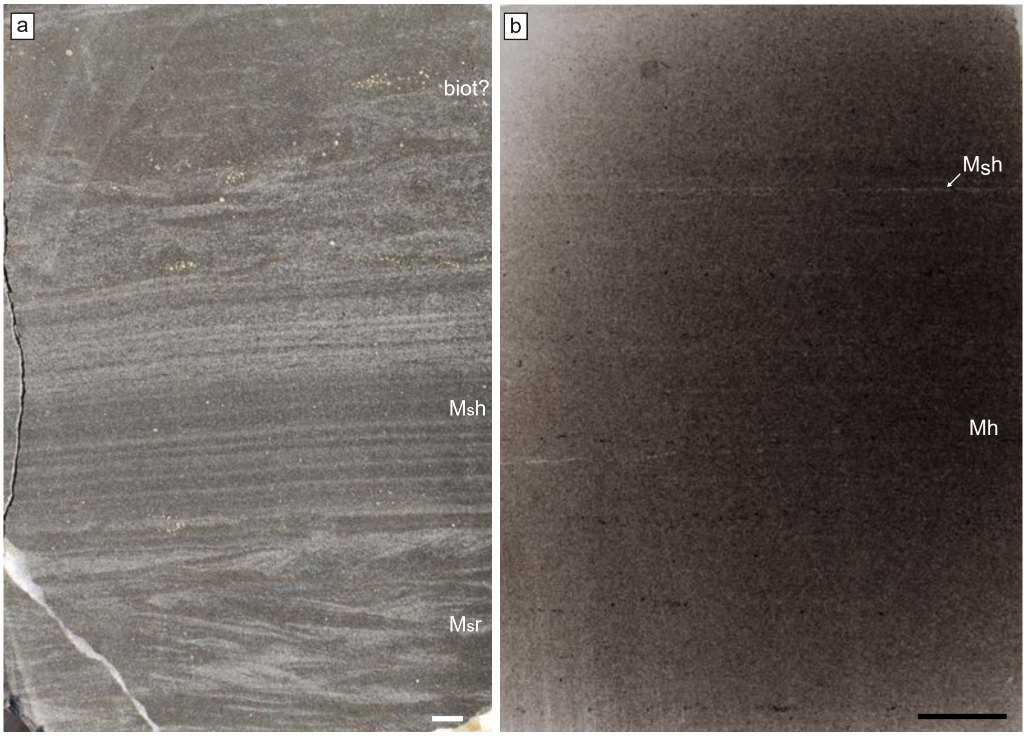

Supplementary Figure 5: Core (a) and a thin section (b) photographs of the hemipelagic deposits. Scale bars are 0.5 cm. a) Local current-ripple cross-laminated (M_sr) and parallel-laminated (M_sh) coarse mudstone, with probable biodeformational structures (biot?). b) Thin section of the hemipelagic deposits, showing parallel-laminated mudstone (Mh) with some intercalated coarse mudstone laminae (M_sh).

	**Fe_{HR}/Fe_T (range)**	**Interpretation**
Shallow delta front	0.15 - 0.33	oxic - some dysoxic
Deeper prodelta	0.26 - 0.39	dysoxic - anoxic

Supplementary Table 1: Fe speciation data (Fe_{HR}/Fe_T) retrieved form Qi et al., 2018. Deposits representing the shallow environment of the Chengjiang Biota are dominantly oxic (the majority shows a value smaller than the boundary between oxic and dysoxic waters at 0.22. Deeper deposits record dysoxic-anoxic conditions. Further details on data interpretation are provided in Qi et al., (2018)[1].

Supplementary References

1. Qi, C., Li, C., Gabbott, S.E., Ma, X., Xie, L., Deng, W., Jin, C. & Hou, X.G. Influence of redox conditions on animal distribution and soft-bodied fossil preservation of the Lower Cambrian Chengjiang Biota. *Palaeogeography, Palaeoclimatology, Palaeoecology* **507**, 180-187 (2018).
